# Assessing the adherence to treatment among patients with cardiovascular diseases in Kermanshah, Iran

**DOI:** 10.34172/hpp.2021.11

**Published:** 2021-02-07

**Authors:** Parisa Janjani, Mohammad Reza Majzoobi, Amir Sanjabi, Mojtaba Movahed, Alireza Rai, Khodamorad Momeni, Reza Heidari Moghadam, Mohammed Rouzbahani, Mhammadreza Saidi, Nahid Salehi

**Affiliations:** ^1^Cardiovascular Research Center, Health Institute, Kermanshah University of Medical Sciences, Kermanshah, Iran; ^2^Developmental Psychology and Clinical Psychology of the Lifespan, Faculty II, University of Siegen, Adolf-Reichwein-Str. 2a D-57068, Siegen, Germany; ^3^Department of Psychology, Faculty of Social Sciences, Razi University, Kermanshah, Iran

**Keywords:** Adherence, Treatment, Determinant, Cardiovascular diseases, Iran

## Abstract

**Background:** The present study aimed to investigate the psychological determinants of adherence to treatment among patients with cardiovascular diseases (CVDs) referring to Imam Ali Hospital in Kermanshah, Iran.

**Methods:** This cross-sectional study was conducted on 227 patients (mean age=58.10, SD = 13.44) with CVDs, randomly selected amongst those admitted to Imam Ali cardiovascular center of Kermanshah in 2018. Data were collected through Meaning in Life Questionnaire (MLQ), the Jefferson Scale of Patient’s Perceptions of Physician Empathy (JSPPPE), the Illness Perception Questionnaire (IPQ), and Adherence to Treatment Questionnaire. The relationships between the criterion and predictor variables were assessed using Pearson correlation coefficient and linear regression (stepwise method) in IBM SPSS Statistics-23.

**Results:** The adherence to treatment was associated with meaning in life (r=0.367), patients’ perceptions of physician empathy (r=0.218), and illness perception (r=-0.238), at the 0.01 level. Meaning in life, patient’s perceptions of physician empathy, and illness perception explained 18.6% of the variance in adherence to treatment. Meaning in life (beta=0.367 and P≤0.001) was the most influential predictor of adherence to treatment. Additionally, there was a significant difference in the score of adherence to treatment by gender (23.46±4.42 for female vs. 24.77±3.53 for male, P = 0.030).

**Conclusion:** The patients’ perceptions of physician empathy, meaning in life, and illness perception were important factors to predict adherence to treatment in patients with CVDs. Gender was a significant predictor of the adherence to treatment.

## Introduction

Nowadays, cardiovascular diseases (CVDs) are the most common cause of death across the world. In recent years, the prevalence of CVDs in less developed countries, like Iran, has had an increasing trend.^1^ CVDs, as deadly diseases, impose high therapeutic costs on human societies. However, there has been no considerable effort to determine the risk factors and the compliance with treatment.^2^ The adherence to long-term treatment among patients with CVDs is a significant issue.^3^ The decrease in the prevalence and therapeutic costs of CVDs can only be achieved through active participation of patients in implementing physicians’ recommendations, referred to as the adherence to treatment.^4^

The Adherence to treatment refers to the extent of patients’ involvement in the behaviors, diet regimens, and treatments recommended by their physicians.^5^ Healthcare professionals believe that poor compliance with medical recommendations may lead to dangerous outcomes such as re-hospitalization. According to Masoudnia, poor adherence to medical recommendations account for 15% of the total re-hospitalization.^6^ The education level, social and cultural status, physicians’ ability to communicate effectively with the patients, physician-patient interaction, health beliefs, the complexity of treatment regimen, treatment period, the side effects of medication, treatment costs and so on are recognized as the main causes of non- adherence with treatment.^4,7,8^

The psychological variables may as well influence the extent of patients’ adherence to treatment. The psychological variables, including the patients’ illness perception, the meaning in life, and the patients’ perception of physician empathy seem to have a significant effect on their adherence to treatment.^9-11^ The patients’ perception of their illness appears to be formed based on information obtained from various sources as well as their own beliefs, which in turn may affect their mental health and ability to adapt themselves to the disease.^12^ The individual, who has suffered from a disease, tries to create a cognitive background of the disease which directs specific behaviors associated with the disease, such as the adherence to treatment and adaptation to the disease. A previous study has indicated that the negative perceptions about disease are correlated with more disability in the future, the reduction of the recovery time, and using more medical services.^13^ Prior study reported that even under the same conditions, patients may have different perceptions of the nature, causes, outcomes, control, and the treatment of their disease.^14^ Furthermore, the happiness and meaning in life affect the adherence to treatment directly and indirectly.^15^ Likewise, patients’ perception of physicians’ empathy as an essential element of quality care improves satisfaction, increases adherence to treatment, and reduces their complaints.^16^

CVDs seem to be the leading cause of mortality in Kermanshah^17^ and, on the other hands, health promotion can enhance adherence to treatment.^18^ Thus, the current study aimed to investigate the psychological determinants of the adherence to treatment among patients with CVDs referring to Imam Ali Hospital in Kermanshah.

## Materials and Methods

### Design and setting

This cross-sectional study was conducted at Imam Ali cardiovascular center in which about two million patients are admitted annually. As the main cardiovascular center in western Iran, Imam Ali Cardiovascular Hospital, with 280 active beds, provides advanced services in the cardiovascular field.

### Study design and sampling

A total of 227 CVD patients were selected to participate in this cross-sectional study. We computed the sample size according to the results of a pilot study, using a confidence level of 95% and a marginal error of 5%, and based on the sample size formula, the estimated sample size was 200. On the other hand, based on a Cochrane table for determining sample size, the minimum being 217. However, considering a possible 5%-10% non-response rate, a sample size of 227 was considered appropriate.

To select the participants, we used a randomization sampling technique: the CVD patients admitted in Imam Ali hospital were randomly selected using Excel randomization.

### Inclusion criteria

Inclusion criteria were age ≥18 years old, having the stable physical status, undergoing coronary artery bypass grafting and/or percutaneous coronary intervention (PCI) and/or coronary angiography, lack of mental medical records, not taking neuroleptic drugs, having mental health certified by a nurse at the hospital, and informed written consent to participate in the study. Subjects who did not sign the informed written consent and/or those on drug abuse were excluded.

### Survey instrument and data collection

*Meaning in Life Questionnaire (MLQ)*

MLQ was developed by Steger et al in 2006.^19^ Firstly, Steger et al made a 44-item scale and then created two factors of meaning in life with 11 items, using exploratory factor analysis. Removing an item, they finally constructed the two factors of meaning in life with 10 items.^20^ The MLQ includes 10 items, scored based on a 7-point Likert scale (strongly agree=7, partly agree=6, agree=5, unsure=4, disagree=3, partly disagree=2 and strongly disagree=1). The reliability and validity of MLQ was confirmed in previous studies. For example, Cronbach’s alpha has been reported between 0.82 and 0.87 for both subscales by Steger et al.^19^ The highest score for the overall MLQ is 70. Interpretation of total score is classified in four groups of very poor perception of the meaning in life (0–17 points), plenty of problems (18–35 points), more positive than negative (36–53 points), and excellent perception of the meaning in life (54–70 points).

*Jefferson Scale of Patient’s Perceptions of Physician Empathy*

Jefferson Scale of Patient’s Perceptions of Physician Empathy (JSPPPE) was developed by Jefferson et al in 2001.^21^ This scale is used to assess the patient’s perceptions of the physician’s empathy. This tool consists of five items, scored based on a 7-point Likert scale (strongly agree=7, partly agree=6, agree=5, unsure=4, disagree=3, partly disagree=2 and strongly disagree=1). Being validated by Faraji,^22^ the current tool indicated a good validity and reliability (Cronbach’s alpha = 0.84). Higher scores reflect the more positive perception of the physician’s empathy. The highest score for the overall JSPPPE is 35. Interpretation of the total score is classified in four groups of very poor perception of the physician’s empathy (0–8 points), plenty of problems (9–17 points), more positive than negative (18–26 points), and excellent perception of the physician’s empathy (27–35 points).

*Illness Perception Questionnaire*

This questionnaire was developed by Weinman et al in 1996.^23^ It consisted of 25 items for assessing the perception of patients suffering from coronary artery disease. This tool consists of 25 items, which are scored according to a 7-point Likert scale (strongly agree=7, partly agree=6, agree=5, unsure=4, disagree=3, partly disagree=2 and strongly disagree=1). The highest score for the overall Illness Perception Questionnaire is 175. Interpretation of total score is classified in four groups of very poor perception of the Illness (0–43 points), plenty of problems (44–87 points), more positive than negative (88–131 points), and excellent perception of the Illness (132–175 points).

*Adherence to Treatment Questionnaire*

The Adherence to Treatment Questionnaire was developed by Ma et al^24^ to assess the adherence to treatment in patients with CVDs. The questionnaire was comprised of 28 items, scored according to a 5-point Likert scale (strongly agree=5, agree=4, unsure=3, disagree=2, and strongly disagree=1). The current questionnaire contains six subscales including diet, exercise, medication, weight control, persuasion, and weight loss. A higher score in this questionnaire means a higher adherence to treatment. The Persian version of the adherence to treatment questionnaire, validated by Mirkarimi et al^25^ had a good validity and reliability (Cronbach’s alpha=0.83). The highest score for the overall Adherence to Treatment Questionnaire is 140. Interpretation of total score is classified in four groups of very poor adherence to treatment (0–34 points), plenty of problems (35–69 points), more positive than negative (70–104 points), and excellent Adherence to Treatment (105–140 points).

To complete the questionnaires, we contacted the supervisors and made appointments to complete the questionnaires. We, then, explained the main objective of the study and obtained patients’ consent to participate. Eligible patients, who signed written informed consent forms, were individually interviewed by two trained interviewers. The interviewers had been trained to ensure that the participants completely understood them.

### Data analysis

The Statistical Package for the Social Sciences (SPSS) (ver. 23.0) was employed for the purpose of data entry, manipulation, and analysis. Quantitative variables were expressed as mean ± standard deviation (SD), and categorical ones as frequencies and percentages. Bivariate Pearson correlation coefficient was utilized to ascertain the magnitude, and direction of the associations between the adherence to treatment with the meaning in life, patient’s perceptions of physician empathy and illness perception. One-way ANOVA and *t* test were performed to compare the adherence to treatment based on socio-demographic variables. Liner regression analysis (stepwise method) was carried out to explain the variation of the adherence to treatment, based on the meaning in life, patient’s perceptions of physician empathy, and illness perception.

## Results

All the 227 patients with CVDs attended the interviews (Table 1). Their mean age was 58.10 (SD 13.44) years. A total of 158 participants (69.6%) were female and 60.8% were Illiterate. Most participants (60.7%) were housewives and about 90% were married. The mean family monthly income was 30 328 200 ± 10 635 800 Rials (Iran’s currency) equal to $150 – with a median income of 30 000 000 Rials.

According to Table 2 which shows the bivariate Pearson correlation between predictor variables (illness perception, patient’s perceptions of physician empathy and the meaning in life) and criterion variable (Adherence to treatment) which were most of them, statistically significant at either 0.05 or 0.01 level. For example, the adherence to treatment was associated with the meaning in life (r=0.367), patient’s perceptions of physician empathy (r=0.218), and illness perception (r=-0.238). Additionally, the meaning in life was significantly related to the illness perception (r = -0.137).

As evident in Table 3, the three predictor variables including the meaning in life, patient’s perceptions of physician empathy, and illness perception were able to explain 18.6% of the variation of the adherence to treatment (F=18.231, *P* = 0.007). The patient’s perceptions of physician empathy had a positive and significant relationship with the adherence to treatment (beta =0.196 and *P* = 0.007). Likewise, there was a negative and significant relationship between the illness perception and adherence to treatment (beta=-0.192 and *P* = 0.002). The meaning in life (beta =0.367 and *P*≤ 0.001) was the most influential predictor on adherence to treatment.

The association between background variables and the adherence to treatment was shown in Table 4, according to which there was a significant difference in the extent of the adherence to treatment in terms of gender. As such, the score of the adherence to treatment in men (24.77±3.53) was higher than that of women (23.46±4.42) (*P* = 0.030).

## Discussion

The purpose of this study was to assess the psychological determinants of the adherence to treatment among patients with CVDs referring to Imam Ali hospital, Kermanshah. Although most studies have focused on the role of social determinants of the adherence to treatment such as quality of life, social support, etc., fewer studies have paid attention to the role of psychological determinants like the meaning in life, the patient’s perceptions of physician empathy and the illness perception among the CVDs patients.

The results of our study illustrated a positive relationship between the adherence to treatment and the meaning in life, and this variable had the capability to predict the adherence to treatment in CVDs. In fact, the meaning in life was the most influential predictor of the adherence to treatment. The results of this study are consistent with those of other researchers. Corless et al reported that there was a significant association between higher life goals and adherence to tuberculosis treatment.^26^ Rosyad et al found a significant correlation between the meaning in life and the adherence of antiretroviral therapy among HIV-seropositive men.^9^ According to Audet and colleagues’ study in 2015, the people with low meaning in life displayed non-adherence in antiretroviral therapy among HIV-seropositives.^27^ A study conducted in Taiwan reported that the meaning in life predicts the self-care behaviors among patients waiting for the heart transplant.^28^ The results of some studies have also indicated that the lack of meaning in life is related to psychological stressors. The lack of meaning in life is associated with the variables such as depression, anxiety, suicide and the drug abuse.^29,30^ Therefore, if life is meaningful, naturally any event however complicated (like chronic illness), will be meaningful. As a result, patients with stronger meaning in life attitude will try to cooperate with physicians to improve their health state (adherence to treatment).

The results demonstrated a positive association between the adherence to treatment and patients’ perceptions of physician empathy. Walsh et al have observed that patients’ perceptions of physician empathy was strongly correlated with patients’ satisfaction, which in turn plays a significant role in adherence to treatment.^10^ Ogle et al found that good physician-patient communication with empathic involvement is related to a better understanding of the instructions given by the doctor and consequently a better adherence to treatment.^31^ Kähkönen et al demonstrated that support from physicians was directly associated with the adherence to treatment among patients with coronary heart disease after PCI.^32^ Actually, physicians increased thanks to their high empathic skills, gain a greater trust from patients, which improve patients’ adherence to treatment. Effective communication between patients and physicians also reduce any maladaptive belief about the health system or physician, which is of significance in patients’ adherence to treatment. Accordingly, physicians’ greater sympathy with the patient results in improved adherence to treatment, which is central to the treatment process.

The results showed a negative relationship between the illness perception and the adherence to treatment. The results of this study are in line with the previous studies. A review study performed by Yalda et al^11^ indicated that in the developed and less developed countries, the patients’ illness perception and an inadequate perception of the therapeutic benefits of medication and procedures play an important role in the adherence to treatment. The illness perception indicates patients’ conceptualization of the disease, which in turn help compliance with the disease and, consequently, improved quality of life.^33^ The findings have shown that the illness perception was one of the most important predictors of low social function, fatigue, anxiety, and low self-esteem directly associated with the adherence to treatment.^34^ When the patient perceived the disease positively, as when they believed that the treatment and medications could improve symptoms, they were more likely to continuously adhere to the doctor’s instructions, which enhances the adherence behavior.

Gender was significantly associated with the adherence to treatment. As such, males reported higher rate of the adherence to treatment than did females. Kähkönen et al indicated that gender was a predictor of the adherence to treatment among patients with coronary heart disease after PCI, and found that the females experienced a higher fear of complications.^32^ In fact, it may be explained by the fact that women, especially those in less developed countries, are mostly dependent on men financially for the continuation of the treatment.

In this study, we encountered four limitations:

1. We used self-reported data to assess the adherence to treatment; such data might be less accurate compared with date gained from observation.
2. The nature of the study design (cross-sectional) did not allow further evaluation of any apparent associations over time.
3. We did not assess all psychological determinants.
4. Our data were derived from a single center; hence, our participants may not be representative of the whole CVDs population.

To best of our knowledge, future studies are required in a larger sample size examining other psychological determinants, e.g., self-esteem, with a longitudinal design. Also, to assess the accuracy of the responses, caregivers and patients’ family members should be asked the same questions about adherence.

## Conclusion

In conclusion, the patients’ perceptions of physicians’ empathy, the meaning in life, and the illness perception were important factors to predict the adherence to treatment in patients with CVDs. Accordingly, to design health programs, more attention needs to be paid on these constructs. Gender was also a significant predictor of the adherence to treatment. Hence, health-care providers and health educators should include gender in further policy making. The access and use of health care programs need to be improved for women, for the improvement in adherence to treatment among women. Finally, future studies are needed to examine other psychological determinants (attitudes, perceived behavioral control, etc.).

## Acknowledgments

The authors would like to thank the Clinical Research Development Center of Taleghani and Imam Ali Hospital, Kermanshah University of Medical Sciences, for their supports, cooperation and assistance throughout the study process.

## Funding

This study was supported by Kermanshah University of Medical Sciences.

## Competing interests

The authors declare that they had no conflicts of interest. In addition, the authors had no financial interest related to any aspect of the study.

## Ethics approval

The protocol of the study was approved by The Research Ethics Board of the Deputy of Research in the KUMS (KUMS.REC.1395.324).

## Authors’ contributions

All authors have, fully or partly, been involved in the concepts and design of the study, collecting the data or preparing the manuscript. In addition all have reviewed the manuscript. PJ, MRM, AS, developed the original idea, the protocol, and study design. MM collected and manage the data. AR, KM, RHM participated in data analyses. MR, MS participated in writing manuscript. NS participated in editing manuscript. All authors provided comments, participated in writing manuscript, and approved the final manuscript.

**Table 1:** Sociodemographic, economical, and clinical information of participants (n=227)

Characteristics		No. (%)
Gender	Female	158 (69.6)
	Male	69 (30.4)
Marital status	Single	3 (1.3)
	Married	224 (98.7)
Education	Illiterate	138 (60.8)
	High school	55 (24.2)
	Diploma	31 (13.7)
	College degree	3 (1.3)
Work status	Jobless or retired	4 (1.8)
	Worker or Farmer (blue collar)	46 (20.3)
	Staff or business (white collar)	25 (11.0)
	Housewife	152 (67.0)
Income	˂ 20×10^6^Rials	22 (9.7)
	20×10^6^ -30×10^6^ Rials	87 (38.3)
	30×10^6^ -40×10^6^ Rials	88 (38.8)
	Over 40×10^6^ Rials	30 (13.2)

**Table 2:** Correlation between different components

**Component**	**Mean (SD)**	**Range**	**X1**	**X2**	**X3**
X1. Illness perception	36.00 (8.80)	15-55	1		
X2. Patient’s perceptions of physician empathy	20.25 (7.23)	5-35	-0.108	1	
X3. The meaning in life	44.39 (7.59)	16-61	-0.137^*^	0.106	1
X4. Adherence to treatment	23.85 (4.20)	11-30	-0.238^**^	0.218^**^	0.367^**^

* Correlation is significant at the 0.05 level (2-tailed). ** Correlation is significant at the 0.01 level (2-tailed).

**Table 3:** Predictors of the adherence to treatment for patients with CVDs

**Variables**	**F**	**R square**	**R square change**	**Adjusted R square**	**Un-standardized** **Coefficients B**	**Std. Error**	**Standardized Coefficients** **Beta**	**t**	**P value**
Step 1									
Meaning in life	34.947	0.134	0.134	0.131	0.203	0.034	0.367	5.912	<0.001
Step 2									
Meaning in life					0.189	0.034	0.340	5.541	<0.001
Illness perception	23.009	0.170	0.036	0.163	-0.091	0.029	-0.192	-3.117	0.002
Step 3									
Meaning in life					0.180	0.034	0.325	5.344	<0.001
Illness perception					-0.084	0.029	-0.176	-2.889	0.004
Patient’s perceptions of physician empathy	18.231	0.197	0.027	0.186	0.096	0.035	0.196	2.714	0.007

Adjusted R square = 0.186, F = 18.231, *P* =0.007

**Table 4:** Comparing the adherence to treatment based on the background variable

**Background variable**	**Subgroup**	**Mean ±SD**	***P*** ** value**
Sex	Female	23.46±4.42	0.030
	Male	24.77±3.53	
Level of Education	Illiterate	23.69±4.46	0.655
	High school	24.00±3.87	
	Diploma	24.52±3.76	
	College degree	22.00±1.00	
Marital status	Single	26.33±2.30	0.286
	Married	23.82±4.32	
Income	˂ 20×106 Rials	23.55±4.39	0.245
	20×106 -30×106 Rials	24.86±3.33	
	30×106 -40×106 Rials	25.04±4.04	
	Over 40×106 Rials	23.30±3.94	
Work status	Jobless or retired	26.00±2.70	0.245
	Worker or Farmer (blue collar)	24.63±3.46	
	Staff or business (white collar)	24.36±4.36	
	Housewife	23.48±4.41	
